# Variable treatment response to lumasiran in pediatric patients with primary hyperoxaluria type 1

**DOI:** 10.1007/s00467-025-06665-w

**Published:** 2025-01-27

**Authors:** Sina Saffe, Katja Doerry, Anja K. Büscher, Matthias Hansen, Melanie Rohmann, Nele Kanzelmeyer, Kay Latta, Markus J. Kemper, Sebastian Loos

**Affiliations:** 1 Department of Pediatrics, Asklepios Klinik Nord Heidberg, Hamburg, Germany; 2 University Medical Center Hamburg-Eppendorf, University Children’s Hospital, Martinistrasse 52, Hamburg, 20246 Germany; 3 Pediatric Nephrology, Children’s Hospital, University of Essen, Essen, Germany; 4 Department of Pediatric Nephrology, KfH-Nierenzentrum Für Kinder Und Jugendliche Beim Clementine Kinderhospital, Frankfurt, Germany; 5 Department of Pediatric Nephrology, Universitätsklinikum Jena, Jena, Germany; 6 Department of Pediatric Nephrology, Medizinische Hochschule Hannover, Kinderklinik, Hannover, Germany; 7 Department of Pediatric Nephrology, Clementine Kinderhospital, Frankfurt, Germany

**Keywords:** RNA interference, Oxalate, Nephrocalcinosis, Kidney stones, Kidney function

## Abstract

**Background:**

Primary hyperoxaluria type 1 (PH 1) is a rare genetic condition due to mutations in the *AGXT* gene. This leads to an overproduction of oxalate in the liver. Hyperoxaluria often causes kidney stones, nephrocalcinosis, and chronic kidney disease. Lumasiran is a recently approved drug that reduces the hepatic oxalate production by mRNA interference.

**Methods:**

In this multicenter study, we evaluated the response to lumasiran treatment in PH 1 patients (*n* = 8) with a median age of 10.9 years (range 1.2–17.9 years), including two patients on hemodialysis. We retrospectively analyzed the reduction of urinary and plasma oxalate levels as well as changes in kidney stone events, nephrocalcinosis, and kidney function.

**Results:**

In patients without kidney failure, the median reduction of urinary oxalate was 64% (range 10–80%) and 71% (61–86%) at 6 and 12 months, respectively. However, only one patient reached urinary oxalate levels within the age-specific normal range. Two patients did not respond to lumasiran and treatment was stopped. In one of the two patients on hemodialysis, the frequency of sessions could be reduced. The only notable side effects were injection site reactions.

**Conclusion:**

There was a variable response to lumasiran in PH 1. Despite a reduction of hyperoxaluria in many patients with PH 1, only one patient reached normal values and 2 of 8 patients did not respond. Regular monitoring of urinary oxalate values and registry data collection seems mandatory to monitor the efficacy and the long-term outcome of PH 1 treated with lumasiran.

**Graphical Abstract:**

**Figure:**
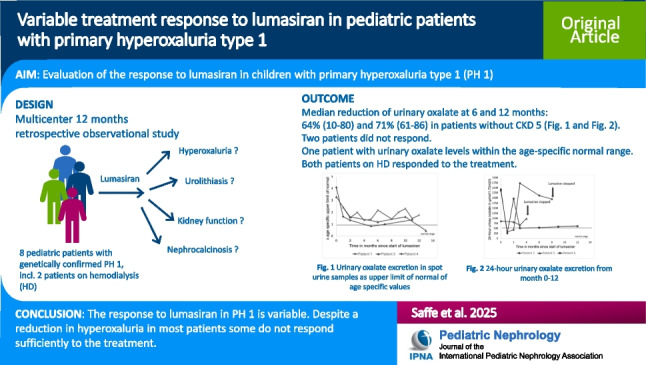
A higher resolution version of the Graphical abstract is available as Supplementary Materials.

**Supplementary Information:**

The online version contains supplementary material available at 10.1007/s00467-025-06665-w.

## Introduction

Primary hyperoxaluria is a rare metabolic disorder that is characterized by a hepatic overproduction of oxalate resulting in hyperoxaluria. Three different types are currently recognized, of which primary hyperoxaluria type 1 (PH 1) is the most common, accounting for 70–80% of all PH patients. The prevalence has been described as 1–3/1,000,000 with an incidence of 1:120,000 [1].

PH 1 is an autosomal recessive genetic disorder and is caused by mutations in the *AGXT* gene, which encodes the enzyme alanine glyoxylate transaminase (AGT). AGT converts glyoxylate to glycine in the liver peroxisome. With reduced or absent enzyme activity glyoxylate is instead converted to oxalate [2]. To date, 190 different mutations have been reported, with p.Gly170Arg, p.Lys12fs, and p.I244T accounting for almost half of all cases [3].

The phenotype is highly variable, ranging from asymptomatic patients who are diagnosed through familial screening to the most severely affected children with infantile PH 1. Most patients suffer from kidney stones and nephrocalcinosis, which over time lead to a deterioration in kidney function that might lead to chronic kidney disease stage 5 (CKD 5). Approximately 1% of children with CKD 5 have PH 1, a proportion that is 10 times higher in countries with increased rates of consanguinity [1]. As kidney function declines, plasma oxalate levels rise and lead to oxalate deposition in extrarenal tissues. This is termed systemic oxalosis and causes devastating end-organ damage, most commonly in the bones and eyes [4, 5].

Various attempts have been made to mitigate the progression of the disease including hyperhydration and treatment with citrate [6, 7]. In addition, about 30% of patients, mostly those with p.Gly170Arg or p.Phe152Ile mutations, respond to high doses of pyridoxine [8–11]. In case of kidney failure, kidney replacement therapy with an intensified hemodialysis regimen alone or in combination with peritoneal dialysis is needed. The only curative treatment to date is a liver transplantation, usually performed as a combined liver and kidney transplantation in those with concurrent kidney failure [12–14].

Due to the known organ shortage and the associated morbidity and mortality of transplantations, new treatment options are urgently needed. Lumasiran was approved in November 2020 for use in patients with PH 1 as it reduces the oxalate production in the liver by RNA interference (RNAi). It is a double-stranded siRNA which targets the mRNA of the gene encoding glycolate oxidase (GO). GO converts glycolate to glyoxylate, the substrate for oxalate synthesis. By reducing the levels of GO through RNAi less glyoxylate and therefore less oxalate is produced [15, 16]. A reduction in plasma and urinary oxalate levels is thought to lead to improved clinical outcomes in patients with PH 1 due to reduced formation of kidney stones, nephrocalcinosis, and deposition of oxalate in other tissues.

As PH 1 is a very rare disease and the experiences with lumasiran treatment are limited to date, it is important that all available data are reported. This multicenter study looked at the real-world outcome of lumasiran treatment in eight pediatric patients at seven centers in Germany.

## Methods

Data of eight pediatric patients treated in seven centers were collected retrospectively. Inclusion criteria were treatment with lumasiran, genetically confirmed PH 1, and age < 18 years at the start of treatment. Follow-up time was 12 months. The dosing and side effects (injection side reaction, fever, rhinitis, headaches, and gastrointestinal symptoms) of lumasiran were documented. The study was approved by the local ethics committee (WF-087/21).

### Evaluation of oxalate levels

In patients with preserved kidney function (eGFR > 15 ml/min/1.73 m^2^), the relative change in urinary oxalate excretion from baseline (month 0) to months 6 and 12 was evaluated. Urinary oxalate was measured either as 24-h urinary oxalate excretion or as oxalate-to-creatinine ratio in a random spot urine sample collected at the clinic visit in patients who had not gained voluntary bladder control or in patients who were unable to reliably collect urine over a 24-h period. A urinary oxalate excretion of ≤ 500 µmol/1.73 m^2^/day was considered normal in the 24-h urine sample, and age-specific normal values according to the 2023 European guidelines were used for the oxalate-to-creatinine ratio in spot urine samples (Supplementary Table 1) [17, 18].

Urinary oxalate was measured by ion chromatography/mass spectrometry (German PH 1 center/Wisplinghoff laboratory, Cologne, Germany), high-performance liquid chromatography (HPLC) (Medical University Hannover (MHH), Germany), mass spectrometry (Synlab, Leverkusen, Germany), or enzymatic assay (Bioscentia, Jena, Germany). All laboratories are certified for these analyses.

In patients on hemodialysis, the relative change in plasma oxalate was analyzed. The plasma oxalate levels were measured by HPLC in the MHH laboratory with a reference range of 3–11 µmol/l in healthy controls. Samples for plasma oxalate were taken in these patients after 2 days without dialysis before the start of the next session.

### Definition of treatment response

Patients who reached urinary oxalate levels within the normal range were considered fully responsive to lumasiran. Those who showed a reduction in hyperoxaluria but did not reach values within the normal range were considered partially responsive. Patients who did not show a reduction in hyperoxaluria were defined as non-responders. In patients on hemodialysis, plasma oxalate levels were compared to pre-treatment values to evaluate the response without the use of a specific cutoff.

### Evaluation of kidney function, nephrocalcinosis, and kidney stones

Further variables were change in kidney function, nephrocalcinosis, and urolithiasis as well as change in dialysis regimen. Kidney function was assessed by eGFR using the revised Schwartz formula and classified according to the KDIGO guidelines into CKD stages 1–5 [19].

Nephrocalcinosis was reported as grade I–III (medullary nephrocalcinosis) and global (corticomedullary nephrocalcinosis) on ultrasound scan [20].

Parents were retrospectively asked to report kidney stone events in the 12 months prior to treatment with lumasiran as well as during the first 12 months of treatment.

### Statistics

Continuous data are presented as median and range.

## Results

The study included four boys and four girls with a median age of 10.9 years (1.2–17.9 years) at the start of treatment. Baseline characteristics and mutations in the *AGXT* gene are presented in Table 1. Three patients had a heterozygous c.508G > A and thus a pyridoxine-sensitive mutation. Seven patients received pyridoxine during their course of disease. Three patients did not respond to pyridoxine leading to discontinuation (patients 1, 4, 7) prior to lumasiran treatment. One patient was not tested for pyridoxine sensitivity by the local center (patient 6)*.* In patients not on dialysis, conservative treatment with hyperhydration and oral citrate was continued when starting lumasiran.

**Table 1:** Baseline characteristics at the start of treatment with lumasiran

Patient	Sex	Genetic mutation	Age at diagnosis (years)	Pyridoxine additionally to lumasiran	Age at start of treatment (years)	NC	Urolithiasis	Kidney function (eGFR, ml/min/ 1.73 m^2^)	24-h UOx (µmol/ 1.73 m^2^/d)^a^	UOx/Cr (mmol/mol)^b^	POx (µmol/l)^c^
1	Female	c.469del, c.508G > A	0.8	No	1.8	Grade III	Yes	> 90	-	454	-
2	Male	c.33dupC, homozygous	0.7	Yes	17.9	No NC	Yes	73	2350	-	-
3	Female	c.584 T > G, homozygous	3.6	Yes	6.2	Global	No	26	-	230	-
4	Male	c.596-1G > C, homozygous	8.3	No	15.8	Grade I	No	> 90	-	248	-
5	Male	c.508G > A, c.846-3C > G	2.6	Yes	15.8	Grade III	Yes	> 90	872	-	-
6	Female	c.847-3C > G, c.203 T > C	4.3	No	4.3	Grade III	Yes	86	2422	-	-
7	Female	c.33dupC, homozygous	0.3	No	1.2	Global	No	HD	-	-	118
8	Male	c.469del, c.508G > A	14.5	Yes	15.5	Global	No	HD	-	-	87

*HD*: hemodialysis; *NC*: nephrocalcinosis; *POx*: plasma oxalate, *UOx*: urinary oxalate; *UOx/Cr*: urinary oxalate/creatinine ratio in the spot urine sample

^a^Normal range < 500 µmol/1.73 m^2^/day [18]

^b^Age-specific normal values according to the 2023 European guidelines [17]﻿

^c^Supersaturation level < 30 µmol/l [21]

At the start of treatment, kidney function was normal in three patients. Two patients had CKD 2 and one patient had CKD 4. Two patients had CKD 5 and were on hemodialysis. Seven patients had nephrocalcinosis and four patients had kidney stones at the start of treatment. Patients 1 and 8 were siblings.

### Reduction of oxalate levels

**Fig. 1:**
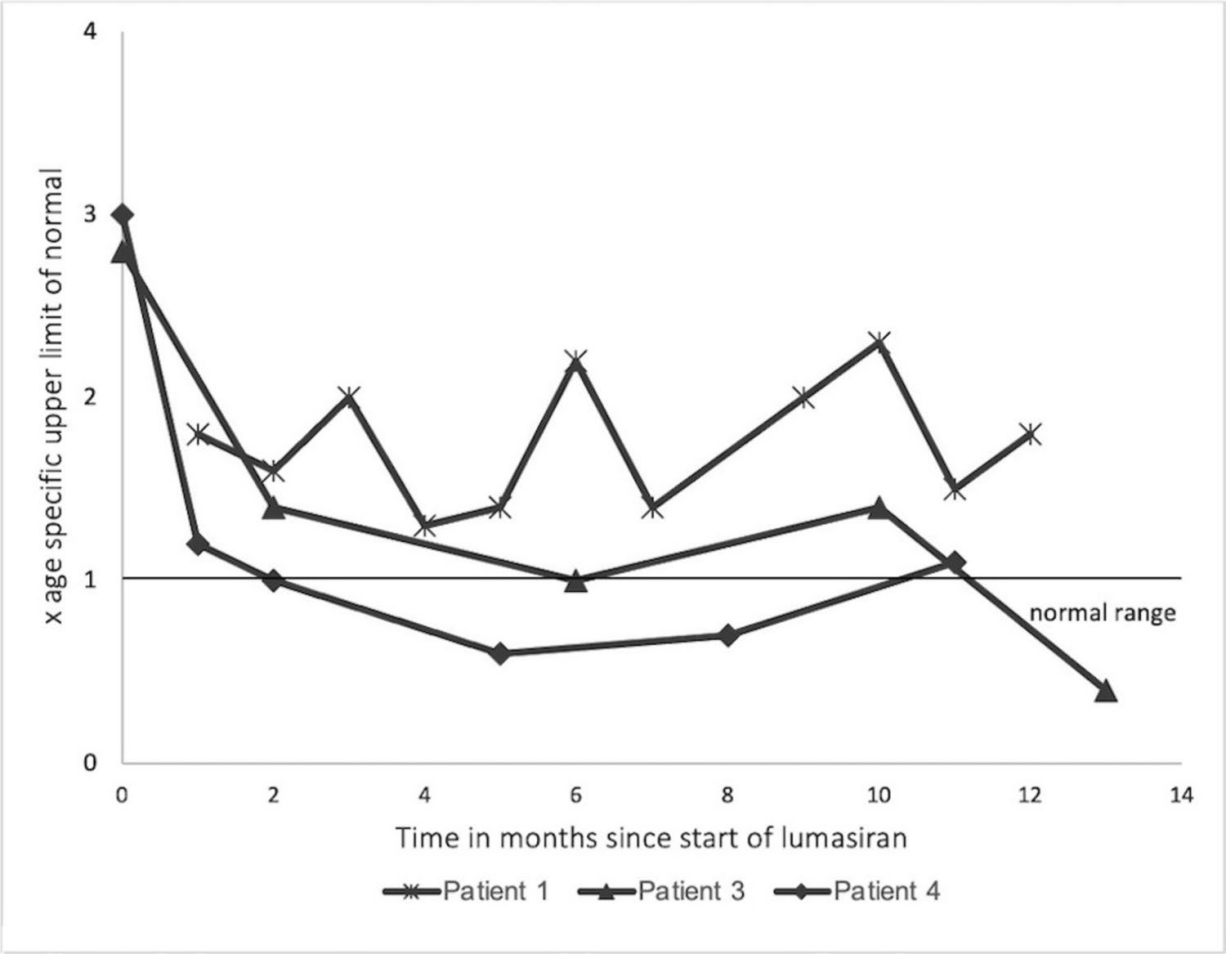
Urinary oxalate excretion in spot urine samples as the upper limit of normal age-specific values [17]

**Fig. 2:**
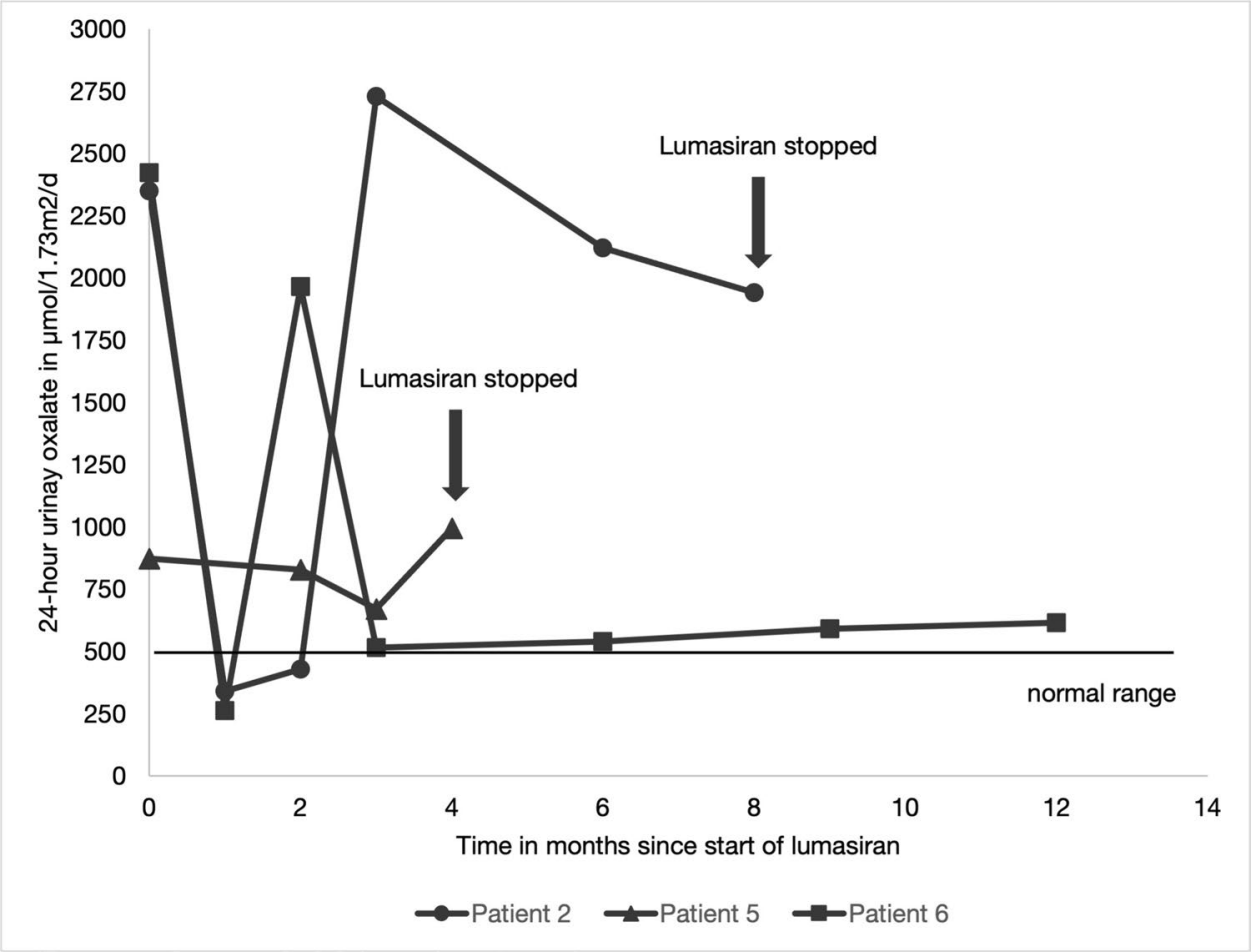
24-h urinary oxalate excretion from month 0 to 12

In patients with preserved kidney function, the median reduction in urinary oxalate was 64% (10–80%) after 6 months and 71% (61–86%) after 12 months of treatment with lumasiran. A 24-h urine sample was collected in three patients, and a spot urine oxalate-to-creatinine ratio was analyzed in the others. The urinary oxalate excretion is shown in Figs. 1 and 2.

After 12 months, only one patient (patient 3) achieved urinary oxalate levels within the age-specific normal range, and two patients (patients 4 and 6) reached levels < 1.5 upper limit of normal (ULN). Patient 1 showed a reduction in hyperoxaluria but did not reach values as low as < 1.5 ULN after 12 months of treatment.

### Patients without response to lumasiran

Two patients, in whom diagnosis of PH 1 was established 17.3 and 13.2 years prior to starting treatment with lumasiran (with a stable degree of hyperoxaluria over the years), did not respond to lumasiran in our cohort: Patient 2 had a rapid initial reduction of urinary oxalate by 82% after 2 months. After the third dose, urinary oxalate levels started to rise again to levels as high as prior to treatment with lumasiran. The dosing frequency was increased to monthly rather than 3-monthly without any effect, leading to a discontinuation of treatment after 8 months (Fig. 2). The urinary glycolate levels in this patient increased from 1.97 to a maximum of 11.55 mmol/d under treatment. The maximal value was documented after 6 months. At this point, hyperoxaluria had already recurred for 3 months. On the last visit before discontinuation of lumasiran, glycolate excretion was 5.75 mmol/d. Of note, 12 months after discontinuation of lumasiran, glycolate excretion was still very high (9.4 mmol/day) as was hyperoxaluria. A genetic analysis excluded a *HAO1* mutation.

Patient 5 showed no response to lumasiran, and the treatment was stopped after 4 months (Fig. 2). The urinary glycolate levels increased from 0.79 to 11.64 mmol/d while receiving lumasiran. Treatment with pyridoxine, citrate, and hyperhydration was continued in both patients.

### Oxalate levels in patients on hemodialysis

Patients 7 and 8 were on hemodialysis at the start of treatment. In patient 8, plasma oxalate levels decreased by 56% after 6 months of treatment. As a result, the frequency of dialysis sessions was reduced from five to three times per week, after which plasma oxalate levels remained < 50 µmol/l.

**Fig. 3:**
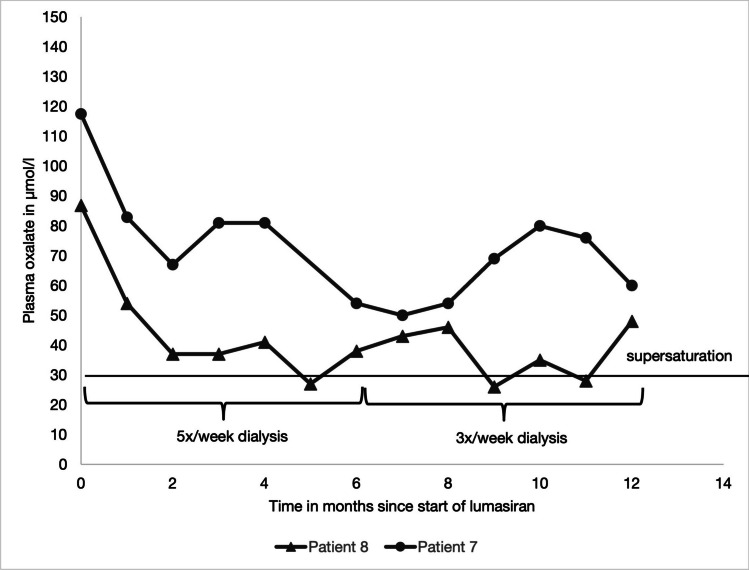
Plasma oxalate levels in hemodialysis patients

Although the plasma oxalate in patient 7 also decreased by 54% after 6 months and by 49% after 12 months of lumasiran treatment, the plasma oxalate levels did not fall below 50 µmol/l. The patient had recurrent bone fractures, serial X-rays showed signs of oxalate osteopathy, and the time per dialysis session was increased despite a reduction in plasma oxalate levels. The plasma oxalate levels of patients 7 and 8 are shown in Fig. 3.

### Urolithiasis and nephrocalcinosis

Four of the patients had kidney stones in the 12 months prior to lumasiran treatment, and patients 1 and 6 showed some improvement after starting lumasiran as reported by the parents. Of the seven patients found to have nephrocalcinosis at the start of treatment, patients 3 and 4 showed an improvement on ultrasound at 12 months. In patient 3, nephrocalcinosis improved dramatically from global to grade I, and in patient 4, nephrocalcinosis was reported to have improved within grade I. No change was observed in the other patients.

### Course of kidney function

The kidney function of patient 3 improved from CKD 4 to CKD 3 after 12 months of treatment with lumasiran. Kidney function remained stable in the other patients. A summary of the outcomes is presented in Tables 2 and 3.

**Table 2:** Outcomes in patients without CKD 5

Patient	UOx 6 months (% reduction)	UOx 12 months (% reduction)	UOx < 1.5 ULN at 12 months	Change in NC at 12 months	Change in urolithiasis events at 12 months	Change in kidney function at 12 months (eGFR in ml/min/ 1.73 m^2^)	Side effects
1	52	61	No	No	Improved	103 to 102	Yes
2	10	Stopped	-	n/a	n/a	73 to 71	Yes
3	64	86	Yes	Global ≥ grade 1	n/a	26 to 32	Yes
4	80	66	Yes	Improved within grade 1	n/a	94 to 96	No
5	Stopped	-	-	n/a	n/a	168 to 116	Yes
6	78	75	Yes	No	Improved	85 to 75	Yes

*NC*: Nephrocalcinosis; *ULN*: upper limit of normal; *UOx*: urinary oxalate

**Table 3:** Outcome in patients with CKD 5

Patient	POx at baseline in µmol/l	POx 6 months in µmol/l (% reduction from baseline)	POx 12 months in µmol/l (% reduction from baseline)	Change in NC at 12 months	Change in urolithiasis events at 12 months	Change in kidney function	Side effects
7	118	54 (54)	60 (49)	No	n/a	Ongoing HD	Yes
8	87	38 (56)	48 (45)	No	n/a	Ongoing HD	Yes

*HD*: hemodialysis; *NC*: nephrocalcinosis; *POx*: plasma oxalate

### Side effects

The only notable side effects were injection site reactions in seven patients, which occurred after 26 of the 56 injections (46%) were given.

## Discussion

This study provides real-world data on the outcome of lumasiran treatment in eight pediatric patients. It highlights the variable effect on hyperoxaluria under treatment with lumasiran including patients with only partial and even non-response.

The median reduction of urinary oxalate in our patients with preserved kidney function was similar to the results obtained in the ILLUMINATE A and B studies and was as high as 71% at 12 months in our cohort. The ILLUMINATE A study was a randomized, double-blind phase 3 trial of lumasiran, which included 36 patients with PH 1, who were ≥ 6 years old and had an eGFR > 30 ml/min/1.73 m^2^. The mean urinary oxalate reduction was 64% after 12 months of treatment with lumasiran. The ILLUMINATE B study looked at 18 patients with PH 1, who were < 6 years and had an eGFR > 45 ml/min/1.73 m^2^. The urinary oxalate excretion was reduced by 72% after 12 months of treatment with lumasiran in this cohort [22–25]. Even though a high median reduction in urinary oxalate was observed in our study, it should be noted that only one of our patients achieved urinary oxalate levels within the age-specific normal range. Similarly, only two patients in the ILLUMINATE B study achieved a normalization in hyperoxaluria after 12 months [24]. It can be argued that any reduction in hyperoxaluria is desirable, especially in patients with extremely high hyperoxaluria. On the other hand, it has been shown that even a mild hyperoxaluria is a risk factor for the progression of CKD [26, 27]. Therefore, patients with only a partial response need careful, long-term follow-up, especially since hyperoxaluria can rise again after a period of response.

A European multicenter study also observed a reduction in urinary oxalate excretion in most patients with preserved kidney function. Similarly to the findings in our study, some patients did not reach urinary oxalate levels < 1.5 ULN, and adaptation of dosing may be needed [28].

Treatment failure leading to the discontinuation of lumasiran occurred in two of our patients and has to date mainly been reported in the adult population [28]. Nonetheless, even in pediatric patients, an insufficient response to lumasiran has been reported. In those case reports, various actions (increasing the dose of lumasiran, adding stiripentol or nedosiran) were taken, and the oxalate levels in the urine or plasma improved thereafter [29, 30].

Both non-responders in this cohort were older than 15 years of age at the start of treatment with lumasiran. The time interval from diagnosis to the initiation of lumasiran was longer than in the other patients (17.3 years and 13.2 years, respectively).

The increase in urinary glycolate levels in patients 2 and 5 during treatment with lumasiran confirms the correct administration and GO inhibition of the drug. Nonetheless, it did not have a sufficient effect on the hyperoxaluria indicating that other pathways could be contributing to raised oxalate levels. This was discussed in a recent paper by Garrelfs et al. and might warrant a trial in these patients with nedosiran, another siRNA which has been recently approved by the FDA and has been shown to reduce hyperoxaluria in patients with PH 1 [31, 32].

The reduction in plasma oxalate levels of 56% and 54% after 6 months in the two patients on hemodialysis is higher than that seen in the ILLUMINATE C study, which looked at the relative change in plasma oxalate in 21 patients of all ages with PH 1 with an eGFR ≤ 45 ml/min/1.73 m^2^. The phase 3 single-arm trial showed a mean reduction in plasma oxalate of 42% in the 15 patients on hemodialysis [33]. Although plasma oxalate levels decreased in both of the dialysis patients in our cohort, no patient achieved plasma oxalate levels below the supersaturation level of 30 µmol/l [21], which is hardly achievable in PH 1 patients on kidney replacement therapy. It has also been shown that elevated plasma oxalate levels are frequently seen even in the non-PH 1 dialysis population [34]. Therefore, a decline in plasma oxalate levels to < 50 µmol/l in patient 8 was considered sufficient to reduce the dialysis frequency from five to three times per week in order to improve quality of life, including school attendance. Nonetheless, a higher oxalate elimination by more frequent hemodialysis would have been desirable prior to transplantation. In the other patient, who had higher plasma oxalate levels (> 100 µmol/l) at the start of treatment, the time per dialysis session was increased due to the earlier described worsening systemic oxalosis. However, it is known that an increase of the time per session above 3 h might not further lower the total oxalate burden [35]. Since bone disease is not immediately improving under lumasiran, it is not surprising that patient 7 had fractures under treatment.

A recent case series of five patients undergoing isolated kidney transplantation and receiving lumasiran treatment showed promising results with all grafts functioning, no nephrocalcinosis, and a urinary oxalate-to-creatinine ratio which was normal or almost normal in four of the five patients. Nonetheless, as there is currently no consensus to guide isolated kidney transplantation versus a combined liver and kidney transplantation in patients with siRNA treatment, both patients were listed for a combined liver and kidney transplantation [36, 37].

Our data show a high variability in treatment response, suggesting that GO inhibition may be less effective in some patients. This hypothesis was studied recently using isotope infusion protocols. These data suggest that three factors, namely less effective inhibition of GO, a high endogenous oxalate production at baseline, and the contribution of precursors other than glycolate, explain the insufficient response or even failure of lumasiran treatment in PH 1 [32].

Two of the seven patients with nephrocalcinosis showed an improvement in nephrocalcinosis on ultrasound during treatment with lumasiran. Both of them had urinary oxalate levels < 1.5 ULN at 12 months, suggesting a correlation between hyperoxaluria and nephrocalcinosis. Various studies have shown that higher urinary oxalate levels as well as nephrocalcinosis are associated with a higher risk of developing kidney failure [26, 38]. Fewer patients showed a change in nephrocalcinosis in our cohort compared to the numbers seen in the ILLUMINATE A, B, and C studies, where 18%, 57%, and 100% of patients showed an improvement at 6 months and 46% and 79% of patients showed an improvement at 12 months, respectively [22–25, 33]. The large variation seen may be due to the high inter-observer variability in grading nephrocalcinosis on ultrasound scan [39]. Furthermore, it may take years before a change in nephrocalcinosis is seen, even after correction of the metabolic defect [40].

Mixed results have been published regarding the changes in kidney stone events during treatment with lumasiran. The ILLUMINATE A study and a case report from Belgium show an improvement in urolithiasis, whereas the ILLUMINATE B study could not confirm these findings [22–25, 41]. In our study, two out of the four patients with kidney stones in the year prior to treatment showed an improvement in urolithiasis according to parental observation.

Kidney function improved in one of our patients and remained stable in the others. The phase 3 clinical trials reported a stable kidney function in their cohorts; a case report from France showed a significant decrease in serum creatinine in an infant after 10 months of treatment with lumasiran [22–25, 29]. However, long-term follow-up data are needed to reliably assess changes in kidney function.

The recommended lumasiran dosing regimen was followed in four of the eight patients (Supplementary Table 2). In patient 2, the dosing interval was shortened to monthly rather than 3-monthly as urinary oxalate levels started to rise to values as high as prior to lumasiran treatment. Patient 7 also received lumasiran more frequently, and in patients 3 and 4, the dosing interval was longer than recommended on one occasion. However, patients 3 and 4 were those that reached normal urinary oxalate levels and urinary oxalate levels < 1.5 ULN.

Overall, the treatment with lumasiran was well tolerated with injection site reactions being the only notable side effects in seven of our eight patients (88%). The proportion of patients with injection site reactions in the phase 3 clinical trials was considerably lower, ranging from 11 to 41% [22–25, 33]. Other known side effects of lumasiran such as fever, rhinitis, headaches, and gastrointestinal symptoms did not occur in our cohort. A termination of treatment in one patient due to pain during injection has been reported in a large cohort of PH 1 patients [28].

The weakness of this study is its retrospective observational nature. Additional data on the oxalate levels prior to starting treatment with lumasiran and oxalate values from multiple samples on a specific time point might be valuable for the evaluation of the treatment effect in the context of intra-individual variation. Furthermore, evaluation by 24-h urine collection may be preferable to spot urine measurements; retrospective evaluation of parental observation for kidney stone events and ultrasound are not the most reliable methods to evaluate stone burden. Nevertheless, these real-world data demonstrate the heterogeneity of the disease and the variable treatment response to lumasiran. It highlights the importance of treating patients with rare diseases in specialized centers not only to facilitate comparison of data. Repeated, long-term measurements of oxalate values are mandatory under such a treatment.

In conclusion, the response to lumasiran in PH 1 is variable. Despite a reduction of hyperoxaluria in many patients, only a fraction achieved normalization and some were even completely unresponsive. This study highlights the need for regular reassessment and monitoring of hyperoxaluria under lumasiran, also in view of the extremely high costs (yearly costs per patient in Germany €31,3940 to €94,1822). Therefore, continuation of other treatment modalities (e.g., hyperhydration, citrate, pyridoxine) seems mandatory, especially in this group.

## Supplementary Information

Below is the link to the electronic supplementary material.

ESM 1 Graphical abstract (PPTX 230 KB)

ESM 2 (DOCX 26.4 KB)

## Data Availability

Datasets are not available because of legal restrictions due to data protection. A request for access can be sent to the corresponding author.
