# Supplementary material for: Variable treatment response to lumasiran in pediatric patients with primary hyperoxaluria type 1
Source: Pediatr Nephrol. 2025 Jan 27;40(6):1929–37. doi: 10.1007/s00467-025-06665-w (PMC12031841; doi:10.1007/s00467-025-06665-w)
Supplement: Supplementary file 1 — Graphical abstract (PPTX 230 KB) [file 467_2025_6665_MOESM1_ESM.pptx]

## Slide 1
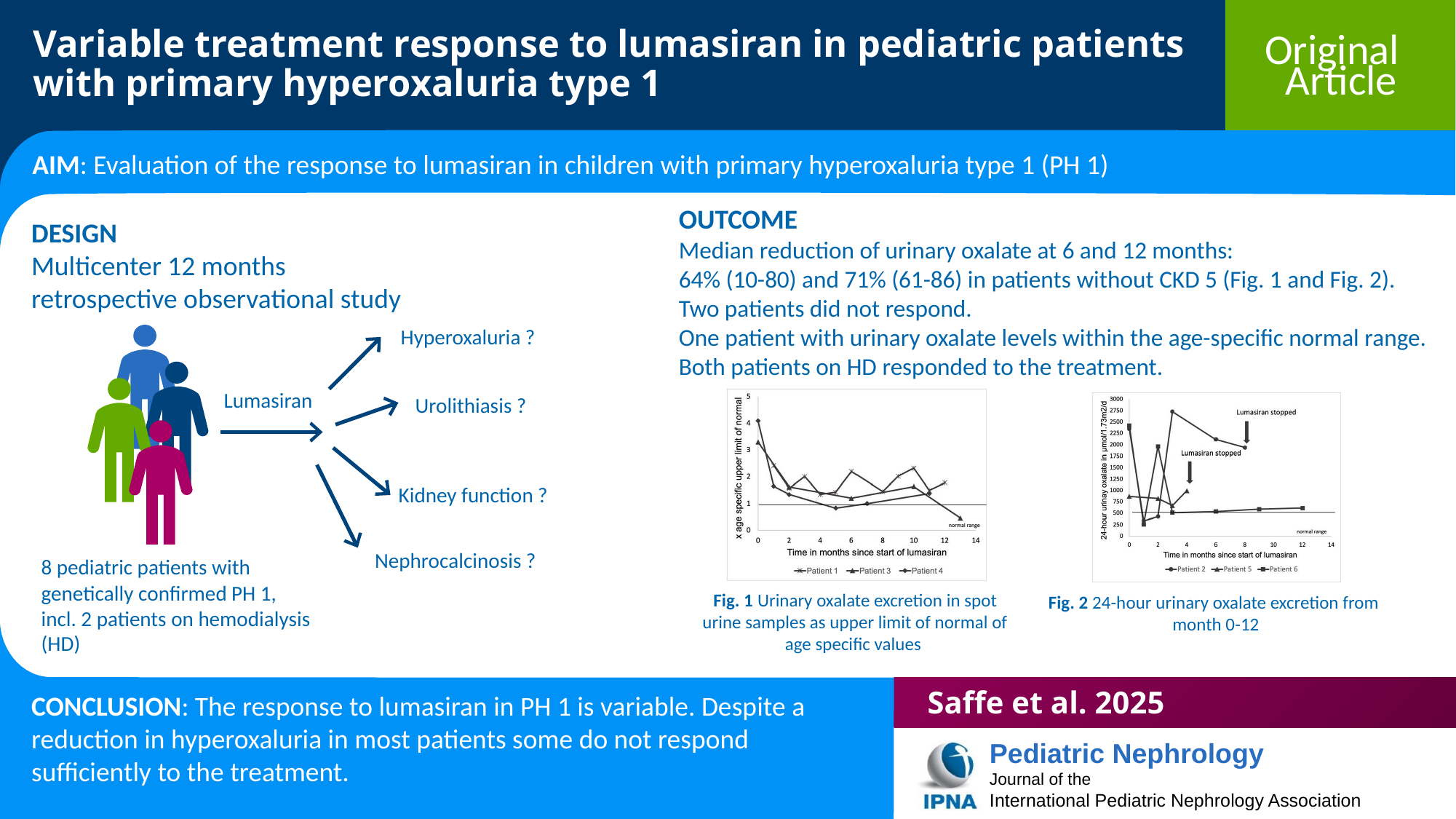

Variable treatment response to lumasiran in pediatric patients
with primary hyperoxaluria type 1
AIM: Evaluation of the response to lumasiran in children with primary hyperoxaluria type 1 (PH 1)
OUTCOME
Median reduction of urinary oxalate at 6 and 12 months:
64% (10-80) and 71% (61-86) in patients without CKD 5 (Fig. 1 and Fig. 2).
Two patients did not respond.
One patient with urinary oxalate levels within the age-specific normal range.
Both patients on HD responded to the treatment.
DESIGN
Multicenter 12 months
retrospective observational study
Hyperoxaluria ?
Lumasiran
Urolithiasis ?
Kidney function ?
Nephrocalcinosis ?
8 pediatric patients with genetically confirmed PH 1, incl. 2 patients on hemodialysis (HD)
Fig. 1 Urinary oxalate excretion in spot urine samples as upper limit of normal of age specific values
Fig. 2 24-hour urinary oxalate excretion from
month 0-12
Saffe et al. 2025
CONCLUSION: The response to lumasiran in PH 1 is variable. Despite a reduction in hyperoxaluria in most patients some do not respond sufficiently to the treatment.
