# Supplementary material for: Variable treatment response to lumasiran in pediatric patients with primary hyperoxaluria type 1
Source: Pediatr Nephrol. 2025 Jan 27;40(6):1929–37. doi: 10.1007/s00467-025-06665-w (PMC12031841; doi:10.1007/s00467-025-06665-w)
Supplement: Supplementary file 2 — (DOCX 26.4 KB) [file 467_2025_6665_MOESM2_ESM.docx]

**Variable Treatment Response to Lumasiran in Pediatric Patients With Primary Hyperoxaluria Type 1**

Sina Saffe, Katja Doerry, Anja K Büscher, Matthias Hansen, Melanie Rohmann, Nele Kanzelmeyer, Kay Latta, Markus J Kemper, Sebastian Loos

**Corresponding author**

Sebastian Loos, MD, University Medical Center Hamburg-Eppendorf, University Children’s Hospital, Martinistrasse 52, 20246 Hamburg, Germany, s.loos@uke.de, Phone: +49-40-7410-20400, Fax: +49-40-7410-51299

**Supp. table 1** Age-specific normal values for urinary oxalate/creatinine ratio in spot urine samples^a^

| Age | UOx/Cr mmol/mol |
| --- | --- |
| 1-6 months | 60-360 |
| 7 months – 2 years | 29-174 |
| 2-5 years | 19-101 |
| > 5 years | < 82 |

^a^ age specific upper limit of normal as 95^th^ centile according to the 2023 European guidelines [17]

UOx/Cr – urinary oxalate/creatinine ratio

**Supp. table 2** Lumasiran dosing (mg/kg) regimen

| Patient | Month 0-2 | Month 3 | Month 4 | Month 5 | Month 6 | Month 7 | Month 8 | Month 9 | Month 10 | Month 11 | Month 12 |
| --- | --- | --- | --- | --- | --- | --- | --- | --- | --- | --- | --- |
| 1 | 6 | 6 | - | - | 6 | - | - | 6 | - | - | 6 |
| 2 | 3 | 3 | - | 3 | 3 | 3 | stopped |  |  |  |  |
| 3 | 3 | 3 | - | - | 3 | - | - | - | 3 | - | - |
| 4 | 3 | - | - | 3 | - | - | 3 | - | - | 3 | - |
| 5 | 3 | 3 | stopped |  |  |  |  |  |  |  |  |
| 6 | 6 | 6 | - | - | 6 | - | - | 6 | - | - | 6 |
| 7 | 6 | 3 | 3 | - | 6 | 6 | - | 6 | 6 | 6 | 6 |
| 8 | 6 | 6 | - | - | 6 | - | - | 6 | - | - | 6 |

**Standard dosing regimen:**

Bodyweight < 10 kg: 6 mg/kg once monthly for 3 doses, 3 mg/kg once monthly beginning one month after the last loading dose

Bodyweight 10-20 kg: 6 mg/kg once monthly for 3 doses, 6 mg/kg once every 3 months beginning one month after the last loading dose

Bodyweight > 20 kg: 3 mg/kg once monthly for 3 doses, 3 mg/kg once every 3 months beginning one month after the last loading dose
